# Risk factors and a prediction model for ASD symptoms in Chinese preschool children

**DOI:** 10.3389/fpsyt.2026.1749880

**Published:** 2026-03-11

**Authors:** Xiaoqi Zhong, Qiyun Jin, Shilin Zhang, Zhijun Liu

**Affiliations:** 1 School of Public Health, Zunyi Medical University, Zunyi, China; 2 Division of Nutrition, Zunyi Maternal and Child Health Care Hospital, Zunyi, China; 3 School of Management, Zunyi Medical University, Zunyi, China

**Keywords:** ASD symptoms, nomogram, prediction model, preschool children, risk factors

## Abstract

**Background:**

The global prevalence of autism spectrum disorder (ASD) is rising, creating an urgent need for practical early screening tools, especially in community and resource-limited settings. This study aimed to identify key risk factors and develop an individualized prediction model for ASD symptoms in Chinese preschool children.

**Methods:**

A cross-sectional study was conducted in 2024, involving 13,641 children aged 3–6 years from 32 kindergartens in Guizhou Province, China. ASD symptoms were screened using the Autism Behavior Checklist. Predictor variables were selected via LASSO regression with 10-fold cross-validation. A multivariable logistic regression model was constructed and presented as a nomogram. Model discrimination was evaluated by the area under the receiver operating characteristic curve (AUC) with bootstrapped 95% confidence intervals (CI). Calibration was assessed using calibration curves and the Hosmer-Lemeshow test, and clinical utility was measured by decision curve analysis.

**Results:**

Among the participants, 324 (2.38%) screened positive for ASD symptoms. Multivariable analysis identified several independent risk factors: lower degree of fondness for the child (OR = 1.53, 95% CI: 1.29–1.81), inconsistency in parenting beliefs (OR = 1.17, 95% CI: 1.06–1.30), poorer sleep quality (OR = 1.55, 95% CI: 1.33–1.80), and a family history of mental disorders (OR = 2.80, 95% CI: 1.81–4.32). Higher parental education (OR = 0.86, 95% CI: 0.78–0.94) and balanced caregiving time (OR = 0.82, 95% CI: 0.76–0.88) were protective factors. The nomogram demonstrated moderate discrimination (AUC = 0.757, 95% CI: 0.731–0.782), was well-calibrated, and provided a net clinical benefit for threshold probabilities between 0.1% and 19.6%.

**Conclusion:**

We successfully developed and validated a practical nomogram that integrates multiple familial and child-level factors for predicting ASD symptoms. This tool exhibits good performance and clinical applicability, offering a valuable approach for early community-based screening of preschool children.

## Introduction

Autism Spectrum Disorder (ASD) is a neurodevelopmental disorder characterized by core deficits in social communication and interaction, alongside restricted, repetitive patterns of behavior, interests, or activities. In recent decades, the global prevalence of ASD has shown a significant increase, with estimates ranging from 0.02% to 3.66%, presenting a serious public health challenge worldwide (1). This trend reflects not only increased public awareness and improved diagnostics but also suggests a historical underestimation of the true disease burden (2). Children with ASD often experience persistent impairments in cognitive, emotional, and social adaptive functioning, with generally poor long-term prognoses, thereby imposing substantial long-term care and economic burdens on families and society. Consequently, developing efficient and precise tools for early identification and risk prediction, building upon existing screening frameworks, is of critical importance for advancing early intervention and improving long-term developmental outcomes.

In China, the burden of ASD is equally severe and exhibits unique epidemiological characteristics. Studies indicate that the prevalence of ASD among children in mainland China is approximately 0.7%, with a marked gender disparity—significantly higher in boys (1.0%) than in girls (0.2%). Furthermore, the disorder’s distribution demonstrates regional imbalance, with a higher reported prevalence in eastern regions (1.2%) compared to central and western areas (3). This pattern likely reflects disparities in healthcare resources, public awareness, and diagnostic capabilities across different regions. Such imbalances in medical resources and diagnostic capacity are particularly pronounced in primary care and rural settings. Research indicates that children with ASD residing in suburban and rural areas experience an average diagnostic delay of at least six months compared to their urban counterparts (4), leading to a large number of children missing the critical window for early intervention. Therefore, research focused on early screening and risk prediction for ASD in central, western, and rural China addresses a particularly urgent public health need.

The exact etiological mechanisms of ASD remain incompletely elucidated, and the prevailing academic view posits that its emergence results from complex interactions between genetic factors and environmental exposures (5). Large-scale epidemiological and genetic studies have confirmed that genetic predisposition constitutes the foremost risk factor for ASD, with heritability estimated at approximately 83% based on Swedish population research (6). Additionally, advanced parental age (7, 8), short interpregnancy intervals (<12 months) (9), maternal infection during pregnancy (10), perinatal complications (e.g., preterm birth, neonatal jaundice) (11), gestational diabetes mellitus (12), and prenatal exposure to air pollutants (e.g., PM_2.5_) (13) have been reported as associated risk factors in multiple studies. However, existing research possesses notable limitations. Firstly, many studies adopt a relatively narrow analytical perspective, failing to simultaneously examine risk and protective factors across multiple dimensions such as family environment, parenting behaviors, child developmental history, and lifestyle habits, thus hindering a comprehensive understanding of ASD risk. Secondly, the majority of research findings remain at the stage of exploring associations with risk factors and have not been translated into individualized risk prediction tools applicable in clinical or community settings, limiting their practical value in early identification practices.

To address these research gaps, this study aims to develop and validate an individualized risk prediction model for the early screening of ASD symptoms, based on large-scale cross-sectional data from preschool children in Guizhou Province, China. We employed an analytical strategy combining variable selection and multivariable regression to construct the prediction model, and visualized the risk using a nomogram. The model’s discrimination, calibration, and clinical utility were systematically evaluated. Ultimately, this study seeks to establish a screening tool that is both scientifically robust and practically convenient, providing a quantitative reference for early identification at the community and family levels in China.

## Study subjects and methods

### Study subjects

This cross-sectional study was conducted in Guizhou Province, China, in June 2024. In collaboration with the Zunyi Maternal and Child Health Care Hospital, we recruited 32 kindergartens through the hospital’s network, encompassing Urban, Suburban, Town, and Rural areas. Family location was classified according to the primary caregiver’s self-report in the questionnaire. The sample’s composition reflects the current urbanization profile of Guizhou Province, showing a relatively high proportion of Urban children while still retaining substantial representation from Rural areas, a distribution characteristic commonly observed in kindergarten-based sampling. Additionally, the proportion of male children in our sample (52.6%) aligned with the provincial sex distribution (51.15% male) reported in the 2023 Guizhou Permanent Resident Population Report. Questionnaires were distributed to primary caregivers via the Wenjuanxing platform, with 13,990 initial responses received. To ensure data quality, two attention-check items were included, and completion time was recorded. Only questionnaires that passed both checks and were completed within a reasonable time were retained; data from children with frequent absences were excluded. Following rigorous quality control, valid data from 13,641 children aged 3–6 years were included, yielding an individual-level valid response rate of 97.5%. The study was approved by the Medical Ethics Committee of Zunyi Medical University (Approval No.: ZMU 2020-2-003), and informed consent was obtained from all participants.

### Study tools

All data were collected by experienced researchers. The questionnaire covered the following dimensions: demographic characteristics, family structure and resources, socioeconomic status, child health and developmental history, parenting styles and family interaction, lifestyle habits and media use, and ASD symptoms.

1. Demographic Characteristics: Family location, Sex, Age of the child.

2. Family Structure and Resources: Number of children in the family, Child’s birth order, Family type.

3. Socioeconomic Status: Highest educational attainment of parents, Father’s occupation, Mother’s occupation, Annual household income per capita.

4. Child Health and Developmental History: Age at first childcare enrollment, Duration of breastfeeding, Term birth status, Mode of delivery, Presence of congenital diseases, Family history of mental disorders, Birth injury or asphyxia.

5. Parenting Styles and Family Interaction:

Proportion of time invested in caregiving: This variable was assessed by asking, “How does the time spent by the father on childcare compare to that spent by the mother?” to quantify the father’s relative contribution. Responses were collected using a six-point ordinal scale ranging from “Much more than mother” to “No involvement”.

Degree of fondness for the child: The primary caregiver’s subjective emotional acceptance was evaluated via the question “How much do you like your child?” using a six-point ordinal scale ranging from “Like very much” to “Dislike extremely”.

Consistency of parenting beliefs: The level of intra-family consensus on parenting goals, rules, and methods was assessed with the question “How consistent are the parenting beliefs between you and your family members?” Responses were measured on a six-point ordinal scale ranging from “Very consistent” to “Very inconsistent”.

Primary source of parenting knowledge: This was a single-item, categorical variable investigating the principal channel through which parents acquired parenting information, skills, and advice.

Duration of parent-child interaction: This metric estimated the average daily cumulative time the child spent in purposeful, focused interactions with parents (e.g., shared reading, playing).

6. Lifestyle Habits and Media Use:

Sleep duration and Sleep quality: Sleep duration referred to the total daily sleep time. Sleep quality was based on the primary caregiver’s overall subjective evaluation in response to “What is your child’s overall sleep quality?”, rated on a five-point ordinal scale ranging from “Very good” to “Very poor”.

Age at first use of electronic devices and Cumulative duration of electronic device use: In this study, electronic devices were explicitly defined as screen-based devices (e.g., television, smartphone, tablet, video game console). Two single-item questions surveyed the starting age for regular use and the average daily cumulative duration of use.

Duration of moderate-intensity physical activity: This variable assessed the daily cumulative time the child spent in moderate- or higher-intensity physical activities (e.g., cycling, brisk walking, running, swimming).

Frequency of sustained sedentary behavior exceeding one hour: This measure evaluated how often, during non-school hours, the child engaged in continuous sedentary behaviors (e.g., reading, screen viewing) for periods exceeding one hour at a time.

7. Autism Behavior Checklist (ABC): The Autism Behavior Checklist (ABC) developed by Krug et al. was used to assess autistic symptoms in children (14). This scale comprises 57 items across five domains: sensation, language, self-care, body movement, and social interaction. Caregivers indicated the presence of each behavior based on the child’s actual situation and scored its significance (1–4 points). A higher total score indicates more severe autistic symptoms. The ABC scale has demonstrated sound psychometric properties in Chinese preschool populations. A recent study based on a Chinese clinical sample reported a screening sensitivity of 79.31% and specificity of 53.13%, supporting the use of a total score ≥ 62 as a valid cutoff (15). Therefore, this study adopted a total score ≥ 62 as the criterion for a positive screen. It should be noted that results from the ABC scale cannot serve as a basis for clinical diagnosis; a definitive diagnosis relies on clinical evaluation based on DSM/ICD criteria and standardized diagnostic tools such as the ADOS. In our sample, the scale showed good internal consistency reliability (Cronbach’s α = 0.898).

### Statistical methods

All statistical analyses were performed using IBM SPSS Statistics 27.0 and R software (version 4.2.0). Categorical variables were described using frequencies (percentages) and compared between the ASD symptom-positive and negative groups using the χ² test. To construct the prediction model, LASSO regression combined with 10-fold cross-validation was first applied to screen potential predictor variables, addressing multicollinearity and preventing overfitting. Subsequently, the selected variables were incorporated into a multivariable logistic regression model to identify independent predictors of ASD symptoms, calculating their odds ratios (OR) and 95% confidence intervals (CI). Based on the final logistic regression model, a predictive nomogram was constructed using the ‘rms’ package in R to facilitate intuitive individual risk assessment. The area under the receiver operating characteristic curve (AUC) was used to evaluate the model’s discriminative ability, and the 95% CI for the AUC was calculated using the Bootstrap resampling method. Calibration was assessed using calibration curves and the Hosmer-Lemeshow goodness-of-fit test. Furthermore, decision curve analysis was employed to evaluate the net clinical benefit of the model across different risk thresholds. All statistical tests were two-sided, with the significance level set at α = 0.05.

## Results

### Sample characteristics of preschool children with ASD symptoms

Among the 13,641 preschool children, 324 (2.38%) screened positive for ASD symptoms based on the ABC scale. The chi-square test revealed that the following variables showed no significant association with ASD symptoms: number of children in the family, child’s birth order, age, duration of breastfeeding, primary source of parenting knowledge, term birth status, presence of congenital diseases, birth injury or asphyxia, duration of moderate-intensity physical activity, and frequency of sustained sedentary behavior exceeding one hour. However, 18 variables, including Family location (χ²=34.511, p =0.002) and Sex (χ²=9.695, p <0.001), demonstrated significant differences between the groups, as detailed in **Table 1**.

**Table 1:** Sample characteristics of preschool children by ASD symptom status.

Variable	Total (proportion)	ASD symptoms (proportion)		χ2 value	*P* value
		No	Yes		
Family location				34.511	0.002
Rural	4679(34.30)	4522(33.96)	157(48.46)		
Town	2547(18.67)	2485(18.66)	62(19.14)		
Suburban	366(2.68)	360(2.70)	6(1.85)		
Urban	6049(44.34)	5950(44.68)	99(30.56)		
Sex				9.695	<0.001
Male	7172(52.58)	6974(52.37)	198(61.11)		
Female	6469(47.42)	6343(47.63)	126(38.89)		
Degree of fondness for the child				56.466	<0.001
Like very much	11630(85.26)	11397(85.58)	233(71.91)		
Like moderately	1771(12.98)	1702(12.78)	69(21.30)		
Like slightly	149(1.09)	136(1.02)	13(4.01)		
Dislike slightly	18(0.13)	16(0.12)	2(0.62)		
Dislike moderately	16(0.12)	14(0.11)	2(0.62)		
Dislike extremely	57(0.42)	52(0.39)	5(1.54)		
Highest educational attainment of parents				67.758	<0.001
Primary school or below	192(1.41)	182(1.37)	10(3.09)		
Junior high school	4206(30.83)	4056(30.46)	150(46.30)		
Vocational school/Technical secondary	1982(14.53)	1920(14.42)	62(19.14)		
Senior high school	1732(12.70)	1708(12.83)	24(7.41)		
Associate degree	2291(16.79)	2253(16.92)	38(11.73)		
Bachelor’s degree or above	3238(23.74)	3198(24.01)	40(12.35)		
Father’s occupation				37.424	<0.001
Unemployed	822(6.03)	790(5.93)	32(9.88)		
Agricultural worker	2523(18.50)	2433(18.27)	90(27.78)		
Manual worker	3608(26.45)	3519(26.42)	89(27.47)		
Business operator	2366(17.34)	2328(17.48)	38(11.73)		
Management/Professional	4322(31.68)	4247(31.89)	75(23.15)		
Mother’s occupation				49.213	<0.001
Unemployed	4290(31.45)	4173(31.34)	117(36.11)		
Agricultural worker	1400(10.26)	1344(10.09)	56(17.28)		
Manual worker	3453(25.31)	3355(25.19)	98(30.25)		
Business operator	1611(11.81)	1592(11.95)	19(5.86)		
Management/Professional	2887(21.16)	2853(21.42)	34(10.49)		
Annual household income per capita				11.746	0.019
< 10,000 RMB	5617(41.18)	5467(41.05)	150(46.30)		
10,000-20,000 RMB	1782(13.06)	1736(13.04)	46(14.20)		
20,000-40,000 RMB	1943(14.24)	1889(14.18)	54(16.67)		
40,000-60,000 RMB	1653(12.12)	1624(12.19)	29(8.95)		
> 60,000 RMB	2646(19.40)	2601(19.53)	45(13.89)		
Family type				23.640	<0.001
Children from two-parent households	11992(87.91)	11735(88.12)	257(79.32)		
Children raised by single mothers	207(1.52)	199(1.49)	8(2.47)		
Children raised by single fathers	266(1.95)	254(1.91)	12(3.70)		
Children from mother-stepfather families	97(0.71)	95(0.71)	2(0.62)		
Children from father-stepmother families	74(0.54)	70(0.53)	4(1.23)		
Children raised by grandparents	1005(7.37)	964(7.24)	41(12.65)		
Age at first childcare enrollment				18.955	<0.001
<1 year	3(0.02)	3(0.02)	0(0.00)		
1–2 years	186(1.36)	185(1.39)	1(0.31)		
2–3 years	2430(17.81)	2397(18.00)	33(10.19)		
>3 years	11022(80.80)	10732(80.59)	290(89.51)		
Proportion of time invested in caregiving				59.336	<0.001
Much more than mother	1192(8.74)	1132(8.50)	60(18.52)		
Slightly more than mother	1440(10.56)	1391(10.45)	49(15.12)		
About the same as mother	2838(20.80)	2780(20.88)	58(17.90)		
Slightly less than mother	3735(27.38)	3679(27.63)	56(17.28)		
Much less than mother	3955(28.99)	3870(29.06)	85(26.23)		
No involvement	481(3.53)	465(3.49)	16(4.94)		
Consistency of parenting beliefs				46.017	<0.001
Very consistent	1627(11.93)	1589(11.93)	38(11.73)		
Mostly consistent	7403(54.27)	7262(54.53)	141(43.52)		
Moderately consistent	2759(20.23)	2697(20.25)	62(19.14)		
Moderately inconsistent	1186(8.69)	1138(8.55)	48(14.81)		
Mostly inconsistent	537(3.94)	510(3.83)	27(8.33)		
Very inconsistent	129(0.95)	121(0.91)	8(2.47)		
Sleep duration				48.964	<0.001
<8 hours	700(5.13)	659(4.95)	41(12.65)		
8–9 hours	5765(42.26)	5613(42.15)	152(46.91)		
10–11 hours	6032(44.22)	5926(44.50)	106(32.72)		
12–13 hours	1054(7.73)	1032(7.75)	22(6.79)		
≥14 hours	90(0.66)	87(0.65)	3(0.93)		
Sleep quality				74.338	<0.001
Very good	4853(35.58)	4770(35.82)	83(25.62)		
Good	6849(50.21)	6710(50.39)	139(42.90)		
Average	1808(13.25)	1719(12.91)	89(27.47)		
Poor	110(0.81)	98(0.74)	12(3.70)		
Very poor	21(0.15)	20(0.15)	1(0.31)		
Age at first use of electronic devices				11.966	0.008
<1 year	783(5.74)	751(5.64)	32(9.88)		
1–2 years	4494(32.94)	4382(32.91)	112(34.57)		
2–3 years	5285(38.74)	5172(38.84)	113(34.88)		
>3 years	3079(22.57)	3012(22.62)	67(20.68)		
Cumulative duration of electronic device use				48.239	<0.001
<1 hour	5844(42.84)	5720(42.95)	124(38.27)		
1–2 hours	5828(42.72)	5698(42.79)	130(40.12)		
3–4 hours	1720(12.61)	1672(12.56)	48(14.81)		
>4 hours	249(1.83)	227(1.70)	22(6.79)		
Duration of parent-child interaction				29.959	<0.001
<1 hour	6506(47.69)	6303(47.33)	203(62.65)		
1–2 hours	6002(44.00)	5901(44.31)	101(31.17)		
3–4 hours	949(6.96)	933(7.01)	16(4.94)		
≥5 hours	184(1.35)	180(1.35)	4(1.23)		
Mode of delivery				9.627	0.017
Vaginal delivery	6904(50.61)	6743(50.63)	161(49.69)		
Cesarean section	6620(48.53)	6465(48.55)	155(47.84)		
Forceps-assisted	48(0.35)	46(0.35)	2(0.62)		
Other	69(0.51)	63(0.47)	6(1.85)		
Family history of ASD or other neuropsychiatric disorders				41.336	<0.001
None	13299(97.49)	13001(97.63)	298(91.98)		
ASD or other neuropsychiatric disorders	342(2.51)	316(2.37)	26(8.02)		

Data are expressed as frequency (percentage). Intergroup comparisons were analyzed using the χ² test; when more than 20% of cells had an expected frequency of less than 5, Fisher’s exact test was employed.

### Variable selection via LASSO regression

**Figure 1:**
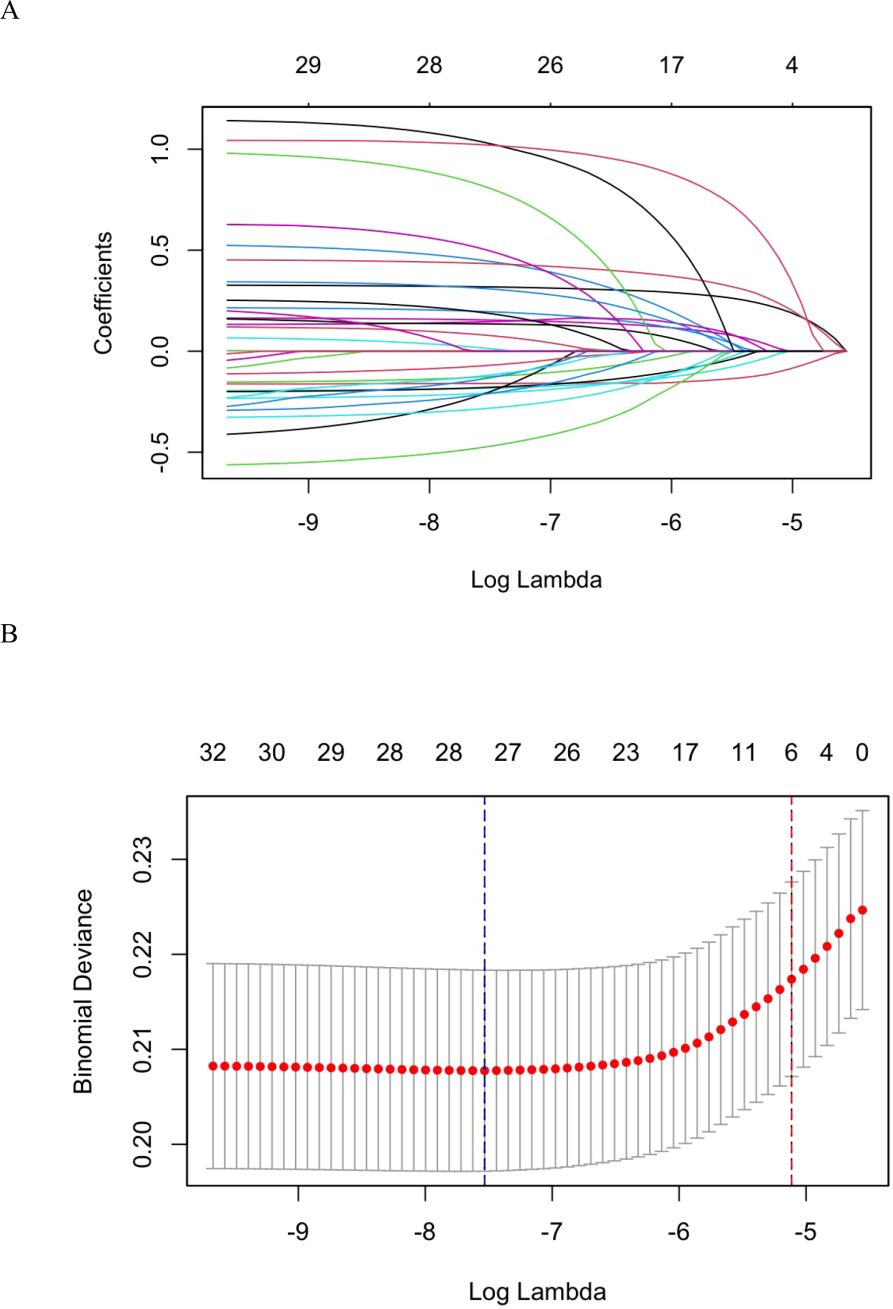
Variable selection based on LASSO regression. **(A)** Coefficient path plot showing the coefficient paths of variables against the log(λ) sequence. **(B)** Cross-validation error plot illustrating the selection process of the optimal λ value in the LASSO regression model via 10-fold cross-validation.

To address multicollinearity and prevent overfitting, LASSO regression was used to screen all 18 candidate predictor variables. The optimal regularization parameter λ was determined via 10-fold cross-validation: λ.min (minimizing cross-validation error) selected 17 original variables, while λ.1se (within one standard error of the minimum) selected 6 original variables (**Figures 1A, B**). To obtain a more parsimonious model with stronger generalizability, λ.1se (λ=0.006014) was chosen as the final criterion. The six variables retained were: Degree of fondness for the child, Sleep quality, Highest educational attainment of parents, Consistency of parenting beliefs, Proportion of time invested in caregiving, and Family history of mental disorders. These variables were subsequently entered into the multivariable logistic regression model to confirm their independent associations with ASD symptoms.

### Prediction model construction

**Figure 2:**
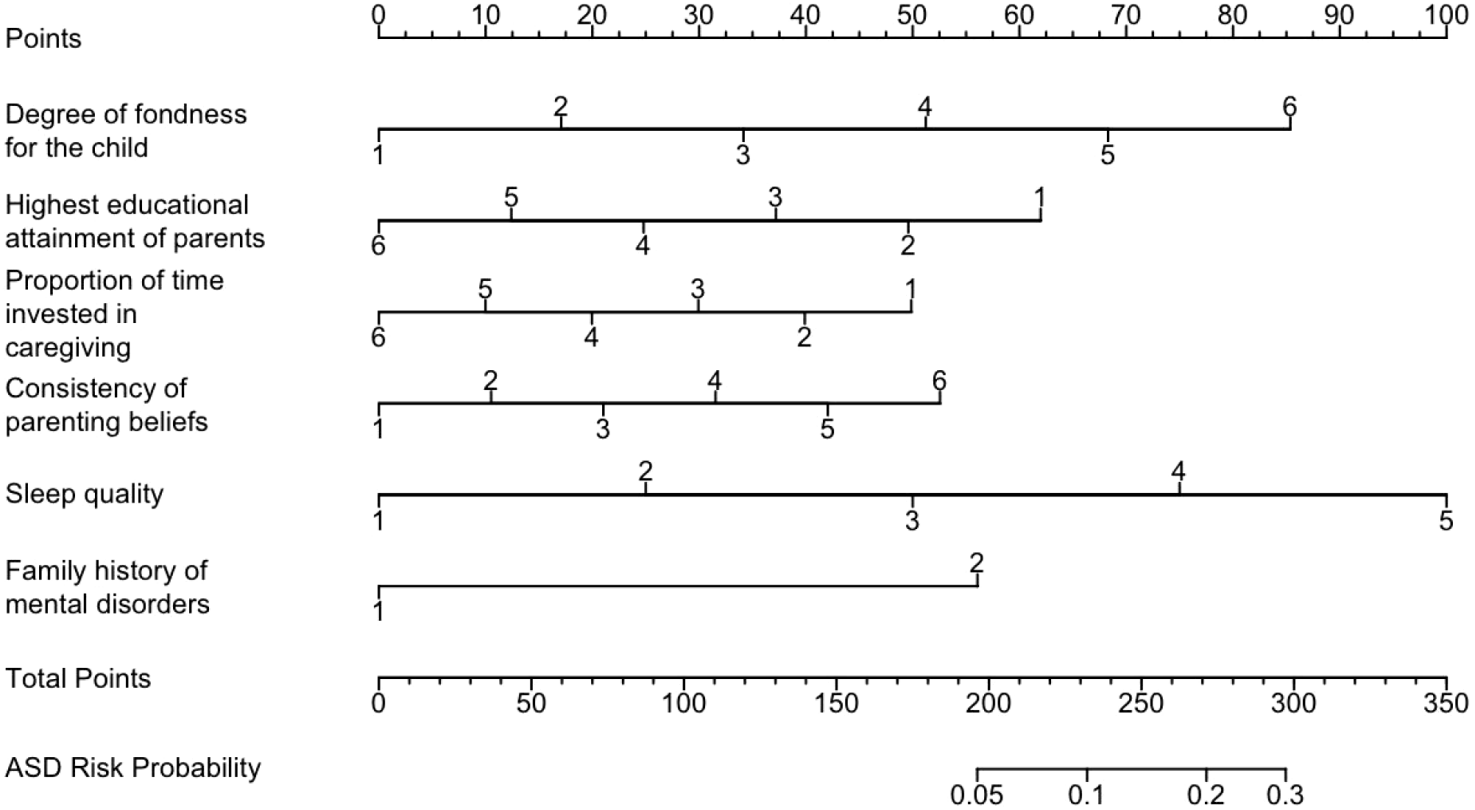
Nomogram for predicting the risk of autism spectrum disorder symptoms in preschool children.

Based on the LASSO regression results, the six selected variables were included in a multivariable logistic regression model to confirm their independent associations. The final model (**Table 2**) identified four risk factors and two protective factors. Family history of mental disorders showed the strongest association (OR = 2.80, 95% CI: 1.81-4.32), followed by poorer Sleep quality (OR = 1.55, 95% CI: 1.33-1.80), a lower Degree of fondness for the child (OR = 1.53, 95% CI: 1.29-1.81), and inconsistency in Parenting beliefs (OR = 1.17, 95% CI: 1.06-1.30). Higher Highest educational attainment of parents (OR = 0.86, 95% CI: 0.78-0.94) and a balanced Proportion of time invested in caregiving (OR = 0.82, 95% CI: 0.76-0.88) were identified as protective factors. All variance inflation factors were ≤ 5, indicating no severe multicollinearity. A predictive nomogram was constructed based on the final multivariable logistic regression model (**Figure 2**), providing an intuitive scoring tool where users can sum the points corresponding to each variable and directly read the individual’s predicted probability of ASD symptoms on the bottom total points axis.

**Table 2:** Multivariable logistic regression analysis of risk factors for ASD symptoms.

Variables	β	SE	Multivariate analysis	
			OR(95% CI)	*P*-value
Unadjusted				
Degree of fondness for the child	0.354	0.066	1.425(1.252,1.622)	<0.001
Highest educational attainment of parents	-0.257	0.039	0.773(0.716,0.835)	<0.001
Proportion of time invested in caregiving	-0.207	0.040	0.813(0.752,0.879)	<0.001
Consistency of parenting beliefs	0.218	0.051	1.244(1.126,1.374)	<0.001
Sleep quality	0.518	0.074	1.679(1.452,1.942)	<0.001
Family history of mental disorders	1.162	0.217	3.198(2.089,4.896)	<0.001
Partially adjusted*				
Degree of fondness for the child	0.346	0.067	1.414(1.240,1.612)	<0.001
Highest educational attainment of parents	-0.172	0.047	0.842(0.768,0.924)	<0.001
Proportion of time invested in caregiving	-0.202	0.039	0.817(0.757,0.883)	<0.001
Consistency of parenting beliefs	0.196	0.051	1.217(1.101,1.345)	<0.001
Sleep quality	0.531	0.074	1.701(1.471,1.968)	<0.001
Family history of mental disorders	1.091	0.220	2.977(1.936,4.579)	<0.001
Fully adjusted**				
Degree of fondness for the child	0.332	0.067	1.394(1.221,1.590)	<0.001
Highest educational attainment of parents	-0.159	0.047	0.853(0.777,0.936)	<0.001
Proportion of time invested in caregiving	-0.203	0.040	0.816(0.755,0.882)	<0.001
Consistency of parenting beliefs	0.166	0.052	1.180(1.065,1.307)	0.002
Sleep quality	0.443	0.076	1.557(1.341,1.809)	<0.001
Family history of mental disorders	1.040	0.222	2.830(1.832,4.370)	<0.001

OR, odds ratio; 95% CI, 95% confidence interval. *Partially adjusted model included: Family location, Sex, Father’s occupation, Mother’s occupation, Annual household income per capita, Family type. **Multivariable adjusted model additionally included: Age at first childcare enrollment, Sleep duration, Age at first use of electronic devices, Cumulative duration of electronic device use, Duration of parent-child interaction, Mode of delivery.

### Prediction model validation

**Figure 3:**
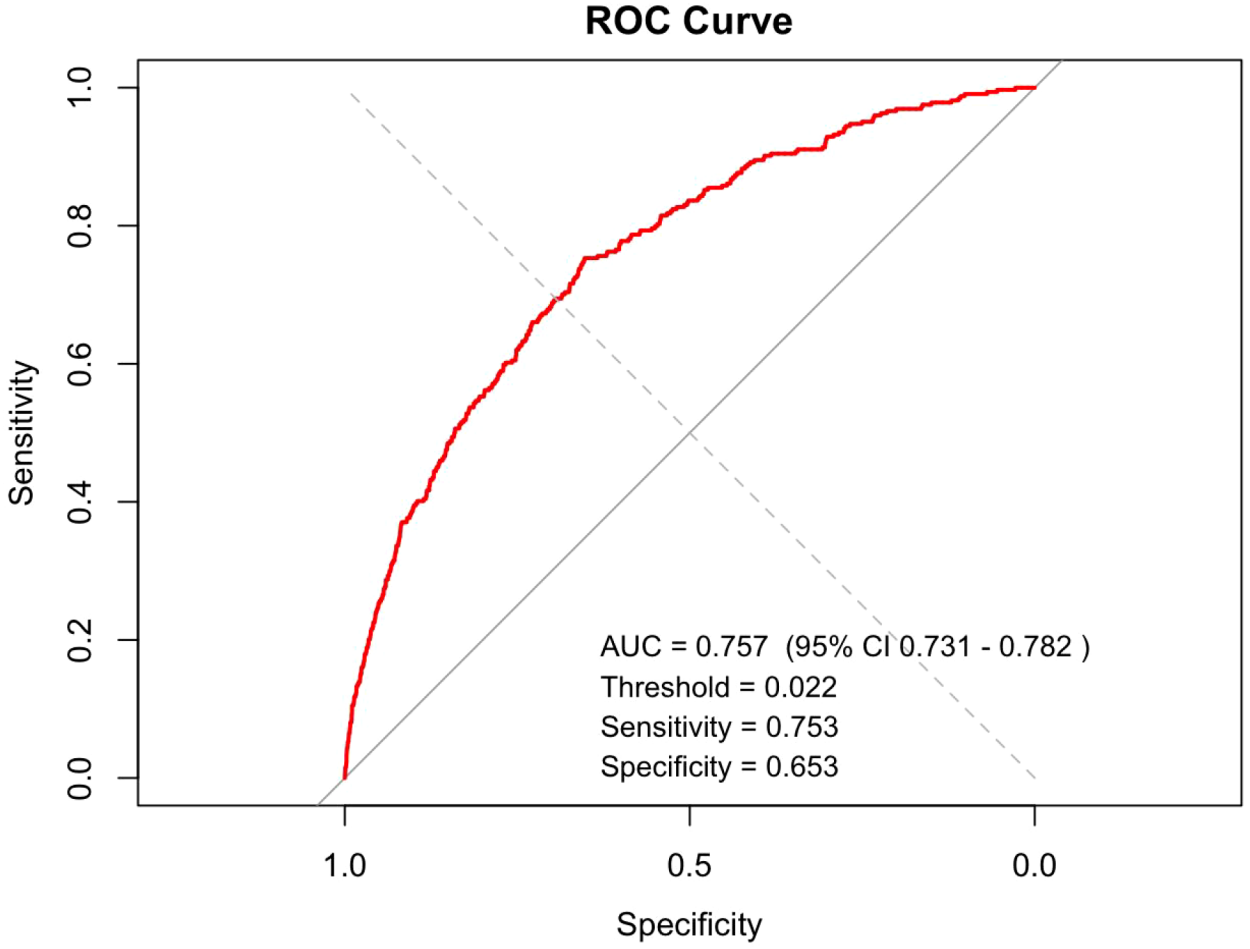
Receiver operating characteristic (ROC) curve of the nomogram prediction model.

**Figure 4:**
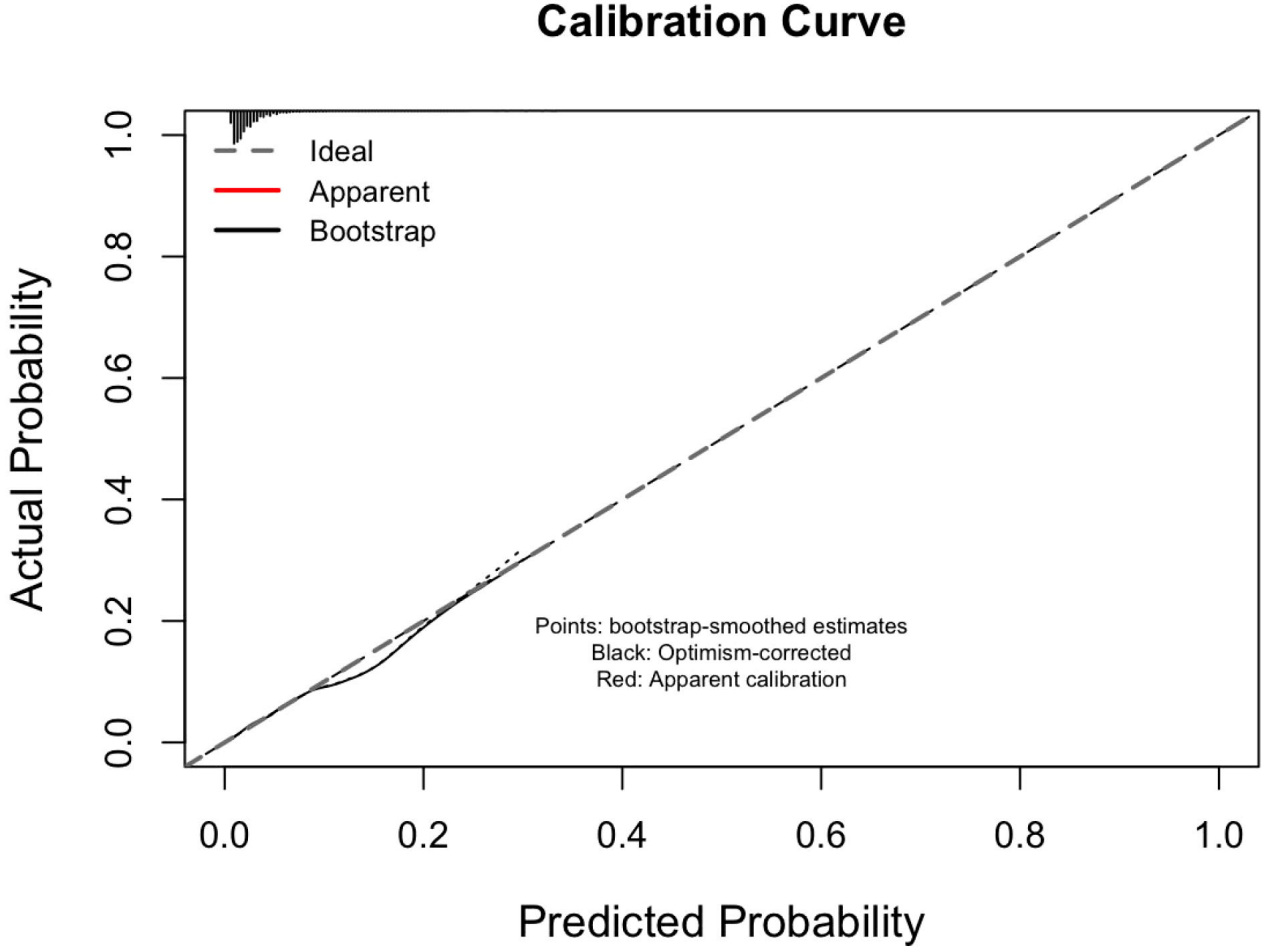
Calibration curve of the nomogram prediction model.

The discriminative ability of the nomogram was assessed using ROC analysis. The results showed an area under the curve (AUC) of 0.757 (95% CI: 0.731–0.782), with an optimal threshold of 0.022 corresponding to a sensitivity of 0.753 and a specificity of 0.653. These findings indicate that the model possesses moderate predictive ability for the occurrence of ASD symptoms in preschool children (**Figure 3**). The calibration performance was further evaluated using calibration curves and the Hosmer-Lemeshow test. The calibration curve demonstrated high consistency between predicted probabilities and actual observed probabilities (**Figure 4**), with a mean absolute error of 0.001 and a 90th percentile absolute error of 0.003, indicating minimal calibration error. The Hosmer-Lemeshow test results supported a good model fit (χ² = 7.6696, p = 0.4664). Collectively, these results suggest that the model exhibits both stable discriminative ability and good calibration.

**Figure 5:**
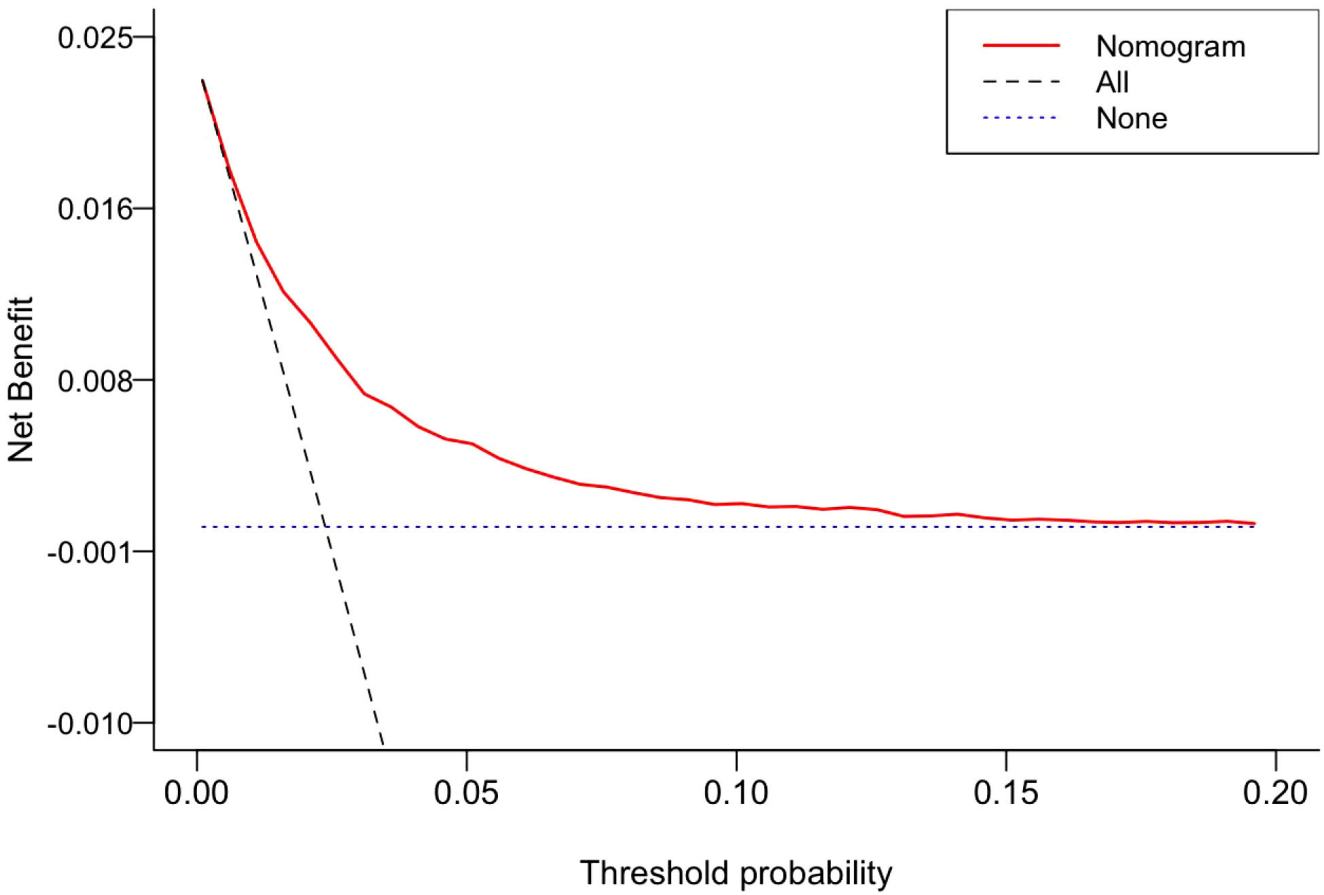
Decision curve analysis (DCA) of the nomogram prediction model. The DCA compares the net benefit of the nomogram model (red solid line) against two reference strategies: “screen all” (black dashed line) and “screen none” (blue dashed line). As shown, within the threshold probability range of 0.1% to 19.6%, applying this model yields a higher net benefit than both extreme strategies, indicating its clinical value within this range.

Decision curve analysis (DCA) was further conducted to evaluate the clinical applicability of the nomogram (**Figure 5**). The model demonstrated a net benefit over the threshold probability range of 0.1% to 19.6%, with an advantage width of 0.195. Within the clinically critical threshold range of 5% to 20%, the model performed consistently across all probability points. The maximum net benefit increase was 0.0085, indicating that at the optimal threshold of 2.6%, screening 1000 children using this model could identify an additional 8.5 cases with positive ASD symptoms. The overall average net benefit (ANB) was 0.000394, further supporting the favorable clinical utility of the model.

## Discussion

To translate the complex risk assessment for preschool ASD screening into a practical tool, this study developed and validated an individualized nomogram that integrates multidimensional predictor variables based on large-scale cross-sectional data. It is important to clarify that the model reveals statistical associations between variables, not causal relationships. Internal validation demonstrated its favorable overall performance, and its design is intuitive and readily applicable to screening practice. Specifically, users can calculate a total score based on the child’s status across the predictor variables and directly obtain the corresponding individualized risk probability from the nomogram. Decision curve analysis further provides a key basis for interpreting this probability, indicating that applying this model yields a clear net clinical benefit when the referral decision threshold is set within the approximate range of 0.1% to 19.6%. This offers empirical support and flexibility for threshold selection in practice. For instance, in primary child health screening in China, an operational threshold could be set by referring to the range associated with higher net benefit identified in this study (e.g., around 2.6%), recommending standardized diagnostic assessment only for children whose estimated risk probability exceeds this value. This strategy can maintain screening sensitivity while optimizing the efficiency of resource allocation, thereby providing a practical tool with both quantitative assessment and decision-support functions to address the challenge of early ASD screening in community settings.

The moderate discriminative ability demonstrated by our model aligns with the common expectations and challenges inherent in early screening within community populations. In public health practice, however, model calibration often carries greater practical significance than discrimination alone. The excellent calibration of our model indicates that its predicted probabilities accurately reflect an individual’s true risk, which is crucial for reliable risk stratification and the optimal allocation of limited screening resources in primary care settings, particularly in resource-limited regions such as Western and rural China. Low-cost, easy-to-administer tools with accurate calibration, like the one developed here, provide critical strategic support for implementing early identification and tiered intervention for ASD.

The association between a lower degree of fondness for the child and an elevated risk of ASD symptoms indicates that children’s social presentation and parental emotional responses are often closely linked within parent-child interactions. Specifically, core symptoms of ASD, such as difficulties in social responsiveness, are frequently associated with challenges children face during such interactions. Moreover, studies have shown that the communication and language skills of children with ASD are significantly correlated with their parents’ emotional states and attitudes (16). Collectively, this evidence suggests that child behavioral characteristics and parental emotional responses tend to co-occur in observed data. Therefore, lower parental emotional investment can be viewed as one component of this complex relational pattern, the stability of which is related to long-term interactive dynamics. This finding implies that, in clinical practice, incorporating parents’ subjective emotional experiences into the assessment framework, in conjunction with focusing on children’s communication abilities, holds important value for a comprehensive understanding and support of parent-child interactions.

Higher parental educational attainment was identified as a protective predictor against ASD risk. This association may result from a combination of environmental, behavioral, and genetic pathways. First, prior evidence supports a statistical link between parental education and offspring ASD risk (17). Parents with higher education generally have greater socioeconomic resources, better access to health information, and increased awareness of developmental issues. These factors are themselves associated with earlier access to developmental assessment and support for children. Second, the resource advantage associated with higher parental education is often correlated with home environments that offer richer cognitive stimulation and more supportive learning conditions. This relationship is particularly pronounced in the role of the mother, as maternal education has been shown to correlate with cognitive and adaptive behavior scores in children with ASD (18). Additionally, although this study focused primarily on observable family factors, genetic intergenerational effects may also contribute to this association, since genetic predispositions related to cognitive and social functioning may be linked to educational attainment as a phenotype (19). Thus, the protective effect of higher parental educational attainment likely represents a complex outcome shaped by both environmental and genetically influenced factors.

A balanced proportion of time invested in caregiving was identified as another key protective factor in this study. We found that ASD symptom risk increased when either parent assumed a disproportionately high or low level of childcare responsibility. This pattern can be explained from the perspective of family system functioning. Existing research indicates that active parental participation and higher self-efficacy in childcare are associated with lower levels of psychological distress (20) and can effectively reduce parenting challenges while improving family dynamics (21). Our findings build upon this existing evidence, suggesting that balanced parental involvement in childcare may create a more stable and supportive family environment, thereby providing more favorable conditions for children’s social-cognitive development. Therefore, in early ASD intervention, beyond focusing on the core symptoms in children, consciously encouraging and guiding both parents to establish balanced childcare responsibilities should be considered a valuable complementary strategy.

Inconsistency in parenting beliefs was identified as an independent risk predictor in this study, with a higher degree of inconsistency associated with an elevated screening risk for ASD in children. When effective communication and consensus between parents are lacking, this typically results in lower consistency in the implementation of intervention strategies and an overall less stable family environment, which may be linked to limitations in the support available for the child’s social-cognitive development. Research indicates that families of children with ASD often exhibit lower family cohesion and reduced emotional expression. Furthermore, parental stress and dysfunctional family states are closely correlated with the severity of children’s symptoms (22). Together, this evidence suggests that inconsistency in parenting beliefs is a family-system characteristic closely associated with developmental risk in children. Therefore, in assessing the risk of ASD symptoms, systematically evaluating family functioning, especially regarding the consistency of parenting beliefs, alongside individual factors, can help identify children and family environments that may require focused attention.

Poorer sleep quality was identified as an independent and significant predictor of ASD symptoms. This study confirmed a significant association between sleep quality and ASD symptom risk, a finding that aligns closely with existing research. Evidence indicates that the prevalence of insomnia among children with ASD ranges from 60% to 86%, and sleep deficiency is closely linked to impaired cognitive function, aggressive behaviors, irritability, inattention, and hyperactivity (23). Clinically, common sleep disturbances in individuals with ASD include insomnia, parasomnias, and circadian rhythm sleep-wake disorders (24), characterized by specific features such as prolonged sleep latency, reduced total sleep time, increased wake time after sleep onset, and decreased sleep efficiency (25). One explanation for this pattern is that the characteristic insistence on routines in ASD may render changes to bedtime particularly distressing, subsequently contributing to delayed sleep onset or insomnia (26). Given the clinical prevalence and amenability to intervention of sleep problems, several studies have explored effective approaches, including the use of melatonin to improve sleep (27), as well as non-pharmacological interventions such as physical activity and relaxation therapies (28). Therefore, incorporating sleep quality assessment into early ASD screening not only aids in identifying high-risk individuals but also provides clear direction for subsequently addressing co-occurring sleep issues to promote the child’s overall health.

Family history of mental disorders emerged as the risk factor with the strongest association in our study, providing substantial support for the significant genetic basis of ASD. In this study, the variable for family history of mental disorders included caregiver reports of a relative’s history of ASD as well as other specified neuropsychiatric conditions such as schizophrenia, bipolar disorder, and major depressive disorder. This finding is consistent with conclusions from large-scale cohort studies indicating that family history of mental and neurological disorders is closely linked to offspring ASD risk (29). Notably, genetic risk for ASD demonstrates broad pleiotropic characteristics, where family histories of various psychiatric disorders, not limited to ASD itself, may constitute genetic risk components for ASD (30). At the risk structure level, parental mental illness types demonstrate distinct, subtype-specific associations with offspring ASD risk: paternal schizophrenia and anxiety disorders are associated with increased ASD risk, while the maternal mental illness spectrum, including schizophrenia, mood disorders, anxiety disorders, and personality disorders, demonstrates broader risk associations (31). This risk pattern is further refined by kinship relationships, with studies showing significantly higher prevalence of mental illness among first-degree relatives (16.9%) compared to second-degree relatives (4%), supporting the value of closer family history as a stronger predictive indicator of risk (32). Based on our findings and existing evidence, systematic inquiry about various neuropsychiatric disorders within three generations on both parental sides, extending beyond ASD alone, holds crucial value for comprehensive assessment of individual genetic risk in clinical genetic counseling and risk evaluation.

Although this study developed a well-performing prediction model based on rigorous statistical methods, its limitations should be interpreted with caution. First, the model has not yet been validated with independent external data; therefore, its generalizability, predictive performance, and calibration in different populations remain uncertain. Second, while the cross-sectional design effectively established predictive associations, it cannot definitively confirm causal relationships between the variables. Finally, several core predictors, including the degree of fondness for the child, sleep quality, and consistency of parenting beliefs, were based on caregiver self-report, which may introduce measurement bias and limit the validity of these constructs. Future research should validate and calibrate this model in multicenter external cohorts, investigate causal pathways through prospective designs, and employ more objective assessment tools.

## Conclusion

This study successfully developed and validated an individualized prediction model for early screening of autism spectrum disorder (ASD) symptoms in preschool children. The model employed LASSO regression combined with multivariable logistic regression to identify core predictors from multidimensional risk factors, and realized intuitive and quantified individual risk assessment through a nomogram. Internal validation demonstrated that the model achieves moderate discriminative ability (AUC = 0.757), favorable calibration, and clinical utility as confirmed by decision curve analysis. With its advantages of rapid administration, cost-effectiveness, and operational ease, this tool provides a reliable quantified basis for promoting early ASD screening within China’s primary child healthcare system.

## Data Availability

The raw data supporting the conclusions of this article will be made available by the authors, without undue reservation.
